# Adverse Drug Reaction Identification and Extraction in Social Media: A Scoping Review

**DOI:** 10.2196/jmir.4304

**Published:** 2015-07-10

**Authors:** Jérémy Lardon, Redhouane Abdellaoui, Florelle Bellet, Hadyl Asfari, Julien Souvignet, Nathalie Texier, Marie-Christine Jaulent, Marie-Noëlle Beyens, Anita Burgun, Cédric Bousquet

**Affiliations:** ^1^ Université Paris 13, Sorbonne Paris Cité, Laboratoire d'Informatique Médicale et d'Ingénieurie des Connaissances en e-Santé (LIMICS), (Unité Mixte de Recherche en Santé, UMR_S 1142), F-93430, Villetaneuse, France Sorbonne Universités, University of Pierre and Marie Curie (UPMC) Université Paris 06, Unité Mixte de Recherche en Santé (UMR_S) 1142, Laboratoire d'Informatique Médicale et d'Ingénieurie des Connaissances en e-Santé (LIMICS), F-75006 Institut National de la Santé et de la Recherche Médicale (INSERM), U1142, Laboratoire d'Informatique Médicale et d'Ingénieurie des Connaissances en e-Santé (LIMICS), F-75006 Paris France; ^2^ Service de Santé Publique et de l’Information Médicale (SSPIM) Department of Public Health and Medical Informatics Centre Hospitalier Universitaire (CHU) University Hospital of Saint Etienne Saint-Etienne France; ^3^ Institut National de la Santé et de la Recherche Médicale (INSERM), Unité Mixte de Recherche en Santé (UMR_S) 1138, équipe 22, Centre de Recherche des Cordeliers, Université Paris Descartes, Sorbonne Paris Cité, F-75006 Paris France; ^4^ Kappa Santé Paris France; ^5^ Centre de Pharmacovigilance, Centre Hospitalier Universitaire (CHU) University Hospital of Saint Etienne Saint-Etienne France; ^6^ Université Paris 13, Sorbonne Paris Cité, Laboratoire d'Informatique Médicale et d'Ingénieurie des Connaissances en e-Santé (LIMICS), (Unité Mixte de Recherche en Santé, UMR_S 1142), F-93430, Villetaneuse, France Sorbonne Universités, University of Pierre and Marie Curie (UPMC) Université Paris 06, Unité Mixte de Recherche en Santé (UMR_S) 1142, Laboratoire d'Informatique Médicale et d'Ingénieurie des Connaissances en e-Santé (LIMICS), F-75006, Paris, Institut National de la Santé et de la Recherche Médicale (INSERM), U1142, Laboratoire d'Informatique Médicale et d'Ingénieurie des Connaissances en e-Santé (LIMICS), F-75006 Paris France; ^7^ Centre de Pharmacovigilance Centre Hospitalier Universitaire (CHU) University Hospital of Saint Etienne Saint-Etienne France; ^8^ Institut National de la Santé et de la Recherche Médicale (INSERM), Unité Mixte de Recherche en Santé (UMR_S) 1138, équipe 22 Centre de Recherche des Cordeliers Université Paris Descartes, Sorbonne Paris Cité, F-75006 Paris France; ^9^ Assistance Publique-Hôpitaux de Paris (AP-HP) Hôpital Européen Georges-Pompidou (HEGP) Department of Medical Informatics Paris France

**Keywords:** pharmacovigilance, adverse drug reaction, Internet, Web 2.0, social media, text mining, scoping review, adverse event

## Abstract

**Background:**

The underreporting of adverse drug reactions (ADRs) through traditional reporting channels is a limitation in the efficiency of the current pharmacovigilance system. Patients’ experiences with drugs that they report on social media represent a new source of data that may have some value in postmarketing safety surveillance.

**Objective:**

A scoping review was undertaken to explore the breadth of evidence about the use of social media as a new source of knowledge for pharmacovigilance.

**Methods:**

Daubt et al’s recommendations for scoping reviews were followed. The research questions were as follows: How can social media be used as a data source for postmarketing drug surveillance? What are the available methods for extracting data? What are the different ways to use these data? We queried PubMed, Embase, and Google Scholar to extract relevant articles that were published before June 2014 and with no lower date limit. Two pairs of reviewers independently screened the selected studies and proposed two themes of review: manual ADR identification (theme 1) and automated ADR extraction from social media (theme 2). Descriptive characteristics were collected from the publications to create a database for themes 1 and 2.

**Results:**

Of the 1032 citations from PubMed and Embase, 11 were relevant to the research question. An additional 13 citations were added after further research on the Internet and in reference lists. Themes 1 and 2 explored 11 and 13 articles, respectively. Ways of approaching the use of social media as a pharmacovigilance data source were identified.

**Conclusions:**

This scoping review noted multiple methods for identifying target data, extracting them, and evaluating the quality of medical information from social media. It also showed some remaining gaps in the field. Studies related to the identification theme usually failed to accurately assess the completeness, quality, and reliability of the data that were analyzed from social media. Regarding extraction, no study proposed a generic approach to easily adding a new site or data source. Additional studies are required to precisely determine the role of social media in the pharmacovigilance system.

## Introduction

Pharmacovigilance is defined by the World Health Organization as “the science and activities relating to the detection, assessment, understanding and prevention of adverse effects or any other drug-related problem” [1]. It comprises postmarketing safety surveillance activities to monitor drug benefit/risk ratios and to identify new potential adverse drug reaction (ADR) signals in real-life conditions. An ADR is defined as “[...] a response to a medicinal product which is noxious and unintended and which occurs at doses normally used in man for the prophylaxis, diagnosis or therapy of disease or for the restoration, correction or modification of physiological function. (WHO, 1972)” [2]. Not all ADRs are identified during clinical trials because of their limited duration and the numbers and types of patients. ADRs need to be followed up after drug approval [3] and, therefore, are burdens for health systems that can potentially lead to hospitalization [4] or death [5].

Pharmacovigilance is mainly based on the spontaneous reporting of ADRs. Initially, only health professionals were allowed to report ADRs. Subsequently, however, a number of studies [6-10] demonstrated the value of patients as reporters. Hughes and Cohen stated that drug user reporting could be a complementary source of knowledge [11]. Currently, a number of countries consider direct patient reporting to be a valuable source in pharmacovigilance [12] and have implemented regulations and solutions for patients’ spontaneous reporting to health authorities. Although patients have increased the number of reporters, ADR underreporting remains a limitation of the current pharmacovigilance system [13-15]. Moreover, other sources for pharmacovigilance are now considered, such as via the secondary use of electronic health records [16-23].

The recent Web 2.0 and social media expansions have been accompanied by a rapid growth in the number of discussions on the Internet regarding drug uses. Social media constitutes a new data source for postmarketing drug safety surveillance [24] and may be of interest in identifying signals because of their high volume and availability.

The use of Internet discussions as an additional data source relies on methods to parse, extract, structure, collect, and organize relevant information from the Web pages for analysis. The use of many sources, the large amount of data, and the heterogeneity of data require multiple steps to obtain analyzable corpora. Methods derived from big data and natural language processing (NLP) need to be considered. Recent works have proposed solutions to address these issues and to standardize the process of extracting information from Web pages in social media.

In addition, questions remain about the quality of information available in users’ Web 2.0 discussions. Whereas electronic health records and health professionals’ ADR reports are structured and well documented, there are no requirements regarding writing and structuring descriptions of pharmacovigilance-related events on social media, and information may be scarce or incomplete.

We performed a scoping review of relevant previously published studies to assess how social media can be used as a data source for postmarketing drug surveillance. This type of literature review aims at providing an overview of the type, extent, and quantity of research available on this topic. Our overview describes the methods used to manage the data from the corpus of Web users’ messages and the obtained results, and identifies potential research gaps and future needs.

## Methods

### Overview

We used the scoping review methodology described by Arksey and O’Malley [25] and further refined by Levac et al [26] and Daudt et al [27]. This methodology divides reviews into six stages: (1) identifying the research question, (2) identifying relevant studies, (3) selecting studies, (4) charting the data, (5) collating, summarizing, and reporting the results, and (6) consultation with stakeholders. Although the sixth stage is optional, we followed the recommendations to consider it a required component.

### Stage 1: Identifying the Research Question

The focus of this scoping review is the use of social media as a new source of data in pharmacovigilance. We use the common definition of social media as media-based or user-generated content. Consequently, we did not consider the news media. To define the search question, we first selected a sample of available publications and found two types: (1) reviews of Web forums conducted by pharmacovigilance specialists on the one hand and (2) technical articles on information extraction approaches authored by computer scientists on the other. In this article, we use the term “identification” to denote the manual process of pulling up social media pages and reviewing them for reports of ADRs. The term “extraction” is used to describe the algorithms that automatically extract ADR information from social media. In the following sections, terms such as “messages,” “social media,” “discussion,” and “page” refer to Web content.

The research question regarding the use of social media or pharmacovigilance is twofold:

1. Theme 1: What is the relevant information for ADR signals that have been issued from social media? The identification theme focuses on the first question and evaluates the information contained in patients’ narrations on social media.

2. Theme 2: What are the methods used to extract information from social media? The extraction theme commits to describing the automated tools and methods that have been used to access structured and valuable pharmacovigilance information.

### Stage 2: Identifying Relevant Studies

**Figure 1:**
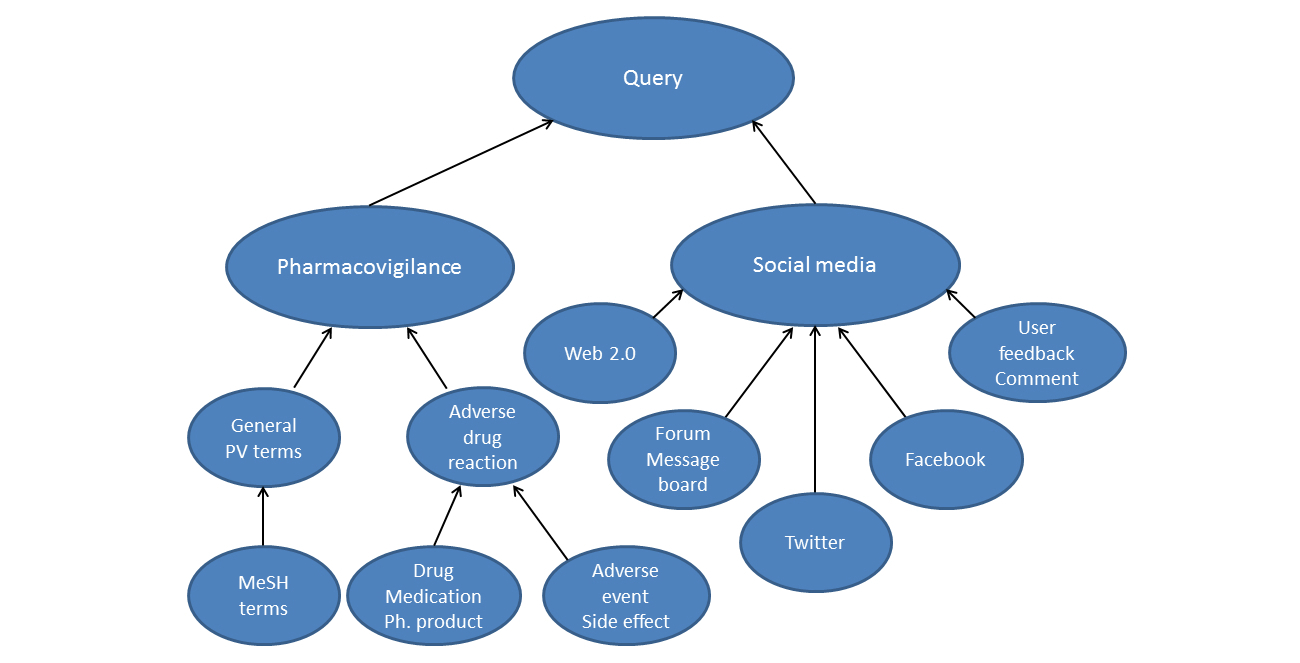
Structure of the search queries.

Two electronic databases—PubMed and Embase—were searched for English and French articles. The PubMed database was searched twice, as follows:

1. With the following keywords (query #1) to investigate the pharmacovigilance and social media dimensions: pharmacovigilance, adverse reaction, adverse event (AE), drug, medication, pharmaceutical product, social media, Web 2.0, social network, Twitter, Facebook, blog, forum, fora, message board, comment, and user feedback. An outline view of this request is presented in Figure 1.

2. Medical Subject Heading (MeSH) terms (query #2)—pharmacovigilance, natural language processing, Adverse Drug Reaction Reporting Systems, and Internet—associated with the following keywords in the title or the abstract: surveillance, Twitter, Facebook, Doctissimo (the main French health-related discussion forum), social media, social network, online health community, online discussion, medical data mining, online, patient forum, and natural language processing.

Query #3 was specially designed for the Embase database based on query #1. The details of the three queries are given in Table 1.

The upper date limit of June 2014 was applied, with no lower date limit, considering that articles published in the early days of social networks could be of interest.

As an iterative process and in accordance with the scoping review methodology, all references from the studies selected in stage 3 were screened, as were all of the publications that cited the selected studies. To broaden the scope of the search, Google Scholar was also used to search for citations.

**Table 1:** Full search strategy for each database.

Database	Query	Query text
**PubMed**		
	Query #1 (keywords)	(pharmacovigilance[MeSH^a^ Terms] OR pharmacovigilance[All Fields] OR ADR^b^[All Fields] OR ADE^c^[All Fields] OR (("adverse reaction"[All Fields] OR "adverse event"[All Fields] OR "side effect"[All Fields]) AND (drug[All Fields] OR medication[All Fields] OR pharmaceutical product*[All Fields]))) AND ("social media"*[All Fields] OR “Web 2.0”[TIAB^d^] OR “Web 2.0”[TIAB] OR "social media" [TIAB] OR "social network*" OR Twitter OR Facebook OR blog OR forum* OR fora OR message board* OR comment* OR (user feedback*))
	Query #2 (MeSH terms)	(((("pharmacovigilance"[MeSH]) OR surveillance[Title])) AND (((((Twitter[Title/Abstract]) OR Facebook[Title/Abstract]) OR Doctissimo[Title/Abstract])) OR (((((((((social media[Title/Abstract]) OR social networks[Title/Abstract]) OR "online health community"[Title/Abstract]) OR "online discussion"[Title/Abstract]) OR medical data mining[Title/Abstract]) OR online[Title/Abstract]) OR patient forum[Title/Abstract]) OR natural language processing[MeSH Terms]) OR "natural language processing"[Title/Abstract]))) OR ((((("Adverse Drug Reaction Reporting Systems"[MeSH]) AND (((((Twitter[Title/Abstract]) OR Facebook[Title/Abstract]) OR Doctissimo[Title/Abstract])) OR (((((((((social media[Title/Abstract]) OR social networks[Title/Abstract]) OR "online health community"[Title/Abstract]) OR "online discussion"[Title/Abstract]) OR medical data mining[Title/Abstract]) OR online[Title/Abstract]) OR patient forum[Title/Abstract]) OR natural language processing[MeSH Terms]) OR "natural language processing"[Title/Abstract])))) OR (((((((((((social media[Title/Abstract]) OR social networks[Title/Abstract]) OR "online health community"[Title/Abstract]) OR "online discussion"[Title/Abstract]) OR medical data mining[Title/Abstract]) OR online[Title/Abstract]) OR patient forum[Title/Abstract]) OR natural language processing[MeSH Terms]) OR "natural language processing"[Title/Abstract])) AND Adverse Drug Reaction Reporting Systems[MeSH Terms])) OR (("Adverse Drug Reaction Reporting Systems"[MeSH]) AND "Internet"[Mesh]))
Embase	Query #3	"pharmacovigilance"/de OR ADR OR ADE OR ("adverse reaction"/de OR "adverse event" OR "side effect"/de AND ("drug"/de OR "medication"/de OR "pharmaceutical product")) AND ("social media"/de OR "Web 2.0":ab,ti^e^ OR "Web 2.0":ab,ti OR "social media":ab,ti OR "social network"/de OR Twitter OR Facebook OR blog OR forum OR fora OR "message board" OR comment OR "user feedback")

^a^MeSH: Medical Subject Heading

^b^ADR: adverse drug reaction

^c^ADE: adverse drug event

^d^TIAB: title and abstract

^e^ab, ti: abstract, title

### Stage 3: Selecting Studies

Four authors (JL, RA, CB, and FB) independently screened the titles and abstracts (when available) of the query results to identify relevant articles. Disagreements about exclusions were discussed until a consensus was reached.

Abstracts were excluded if they met at least one of the following criteria:

1. Not related to drugs.

2. Not related to ADR reporting or ADR detection (eg, efficacy or effectiveness of a study’s design).

3. Not related to patients' reporting (eg, a safety study in animals, signal detection on a pharmacovigilance database).

4. No, or insufficient, results on the use of social media.

5. The study was a review.

6. Soliciting reporting: the study used data from a source where patients were asked to report the ADRs. Patients’ behavior is different depending on whether they are asked to report ADRs or they describe ADRs spontaneously without knowing that there may be further analysis of what they write [28].

7. Editorial: the study did not encompass a result but was an expression of the author's opinion about the usefulness of social media as a new source of knowledge for pharmacovigilance.

### Stage 4: Charting the Data

Two pairs of reviewers independently identified a set of characteristics that could be used to describe the articles in each theme (FB and HA for theme 1, ie, identification; JL and RA for theme 2, ie, extraction). In addition to the basic elementary metadata, a number of characteristics were recorded for each theme. They are listed in Table 2.

The reviewers independently extracted data from the articles that were assigned to them.

**Table 2:** Article characteristics overview.

Characteristics	Theme 1	Theme 2
Year of publication	✓	✓
Language used in the studied texts	✓	✓
Type of data source, for example, forums or Twitter	✓	✓
Presence of an anonymization step	✓	✓
Volume of data analyzed	✓	✓
List of studied drugs	✓	✓
Coding ADRs^a^ (medical lexicon)	✓	✓
Keywords the authors used to identify sources or posts of interest	✓	
Use of semiautomated processes (mixed methods)	✓	
Main results	✓	
Whether reported ADRs were highly informative or not	✓	
Seriousness of reported ADRs	✓	
Reference source was used for comparison with reported ADRs	✓	
Identification of potential unexpected ADRs or unexpected frequency of known ADRs	✓	
Analysis of the influence of other media, for example, television, radio, or the press, as a potential cause of increased ADR reporting in social media	✓	
If the authors mentioned the use of a crawler		✓
Implemented methods of preprocessing		✓
Lay language lexicon or tools used		✓
Authors attempted to identify the relationship between the drug and the event		✓
Authors used a machine-learning approach		✓
Evaluation of the extraction methods with metrics		✓
Comparison with external pharmacovigilance databases		✓
Whether the system enabled evaluating the unexpectedness of any extracted ADRs		✓

^a^ADR: adverse drug reactions

### Stage 5: Collating, Summarizing, and Reporting Results

This work aimed to describe the methods and the results of the two themes. The results for theme 1, “identification,” were related to studies based on the manual search and are presented in terms of methods and quality of data. The second theme, “extraction,” was related to the studies that promoted an automated approach to extracting information from raw data. We summarized the “methods” sections of this last set of studies to describe each step of the methods presented.

### Stage 6: Consultation

Following Daubt et al’s recommendation [27], the research was multidisciplinary and multi-professional. The overall expertise covered pharmacovigilance, pharmacoepidemiology, public health, medical informatics, statistics, and data mining.

This helped us identify additional expectations regarding pharmacovigilance and social media, such as misuses, counterfeit drugs, drug-drug and food-drug interactions, and ADRs in specific populations such as pregnant women. It also permitted us to identify potential stakeholders—health care professionals, regulatory agencies, pharmaceutical companies, and patients—and establish the necessity of measuring the impact of mining social media, the interest in integrating this approach in a practical way in addition to classical reporting systems, and how we can be confident about the findings.

## Results

### Overview of Results

**Figure 2:**
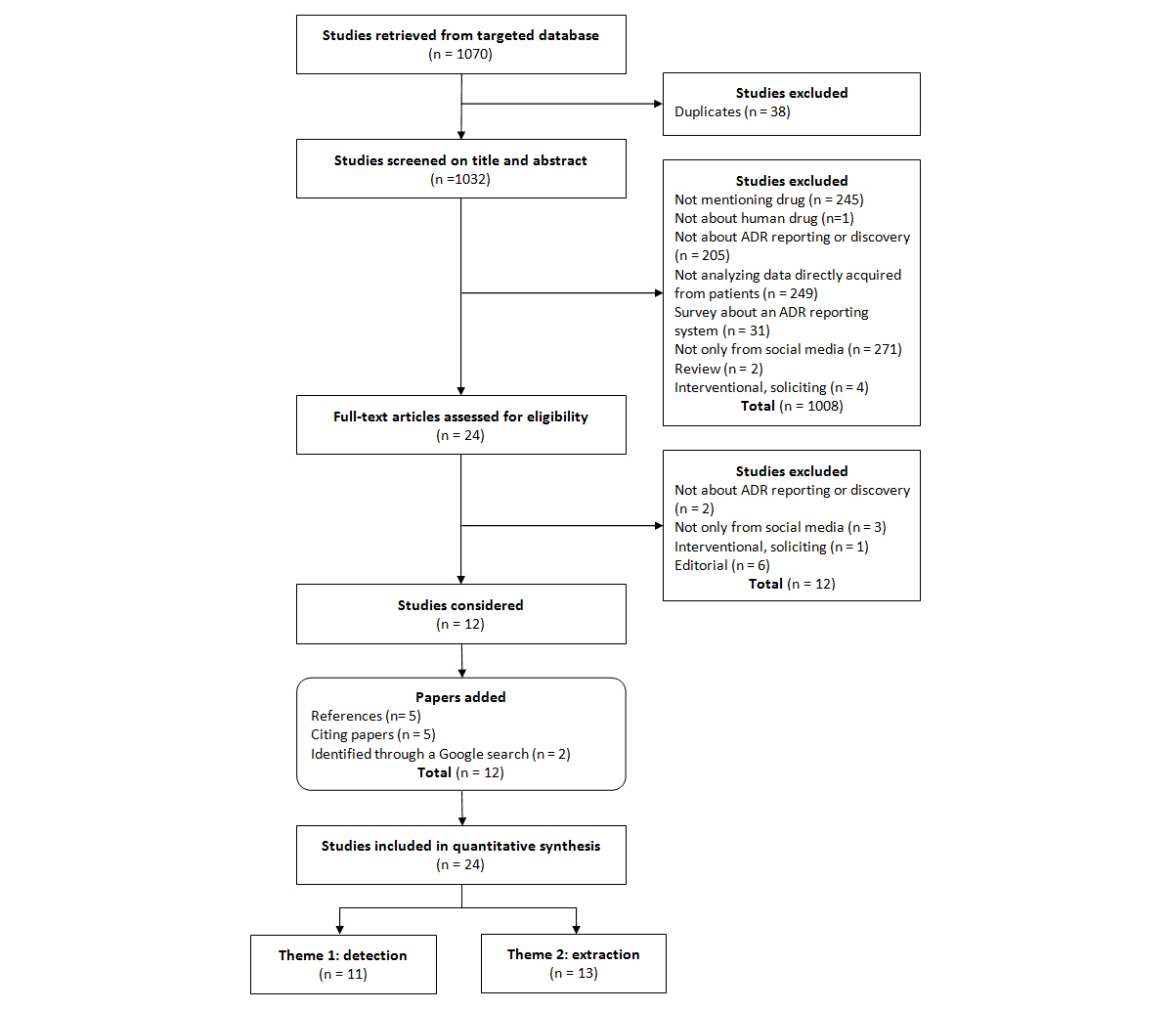
Flowchart of our mapping process and study selection.

Figure 2 depicts the full review process and shows the number of citations excluded at each step.

A total of 1032 publications were identified in PubMed and Embase after duplicates (n=38) were removed. After applying the exclusion criteria to the titles, abstracts, and full texts, 11 citations were relevant to the research question at the end of the screening process (stage 3).

An additional 2 publications were added based on our personal previous knowledge, and 11 studies were identified by the references cited in the publications that were initially selected or by checking other articles that cited these publications on Google Scholar—7 via references and 4 via citing papers.

A total of 24 publications were finally included in the chart process. Of these, 11 (46%) were analyzed for theme 1 (identification) [11,28-37] and 13 (54%) for theme 2 (extraction) [38-50]. The detailed results of charting the data are displayed in Multimedia Appendix 1 and Multimedia Appendix 2.

### Theme 1: Identification

#### Overview

A total of 11 studies described a manual (or mixed) approach for identifying drug-ADR pairs in patients’ narratives that were posted on social media. The majority of these studies were performed in the United States (6/11, 55%) or in France (3/11, 27%). Of these 11 studies, 4 (36%) were published in 2014 and 2 (18%) before 2010—in 2007 [36] and 2009 [34].

In 3 out of the 11 (27%) studies, the authors used the term “adverse event” rather than “adverse drug reaction” to refer to problems reported by patients in social media [30,32,35]. Pages et al justified this in their methodology by stating that the events reported by patients “were not analyzed by health professionals to assess the causal relationship” with the drug [35].

Table 3 details the steps identified in the studies to conduct the manual analysis: (1) selection of data sources, (2) data collection, (3) identification of drug-ADR/AE pairs, and (4) results evaluations.

**Table 3:** Main steps for identifying adverse drug reactions from social media.

Step	Description
Step 0: Selection of data sources	This step consists of identifying and selecting the most relevant websites to answer the research question. They can be identified using a combination of keywords (eg, generic or brand-name drug, disease, ADR^a^/AE^b^) in Web search engines.
Step 1: Data collection	Potentially relevant patient narratives or posts are identified by entering keywords into the search engine hosted by the selected websites (manual identification only) or using a semiautomated process. Data may be imported into software (after anonymization) with the aim of additional analyses.
Step 2: Identification of drug-ADR/AE pairs	The manual identification of drug-ADR/AE pairs is performed by reading the patients' narratives or posts that were initially collected.
Step 3: Results evaluation	This step consists of manually evaluating the frequency and the seriousness of the ADRs or AEs that were identified in patients' narratives or posts. The results can be compared, after coding, with those of other sources (Summary of Product Characteristics [SPC], clinical trials, pharmacovigilance databases, or literature) to identify potential new ADRs or an unexpected frequency of a known ADR.

^a^ADR: adverse drug reaction

^b^AE: adverse event

#### Analyzed Data Sources

The main data source was online forums. Three authors also reported on the analysis of Tweets or blogs [29,30,32]. The selected websites are often devoted to consumers' health. Patients’ comments, mostly written in English, were identified by keywords (eg, brand-name and generic drugs, diseases, ADR) in the search engine hosted by the selected websites. In 2 of 11 (18%) studies, a hybrid (semiautomated) process was performed to identify potentially relevant posts [30,33].

The volume of analyzed data varied according to the studies—from 96 comments [31] to 61,401 Tweets [30]. Most often (ie, 8/11, 73%), the studies analyzed hundreds of narratives or posts.

#### Scope of the Surveillance

Out of 11 studies, 9 (82%) were designed to identify all of the ADRs/AEs that were potentially associated with one or more preselected drugs. Among these 9 studies, 7 (78%) focused on a class of drugs (eg, statins [31] or antineoplastic [33,35], psychotropic [10], or antiparkinsonian agents [36]), a recently marketed drug (eg, dabigatran [37]), or a drug that was removed from the market for pharmacovigilance reasons (eg, benfluorex [28]). Out of the 11 studies, 1 (9%) focused on two specific life-threatening ADRs—Stevens-Johnson syndrome and toxic epidermal necrolysis—and aimed at identifying any potentially associated drugs [29]. Another (1/11, 9%) was designed to identify and analyze predefined drug-AE pairs [34].

The rate of detected ADRs or AEs among the analyzed patients' comments varied according to the studies and was difficult to compare considering the methodological heterogeneity. Whereas Kmetz et al found a relatively low rate of reported AEs among patients' posts containing mentions of targeted drugs—0.3% of all brand mentions and 3.3% of brand mentions that contained side effects keywords [32]—Butt et al identified a large number of Internet descriptions for two rare and serious ADRs [29].

The informativeness of patients’ comments was more or less evaluated in 8 of the 11 (73%) selected studies. In 5 of these 8 (63%) publications, information concerning patients’ characteristics (ie, age, gender, and medical history), suspected drugs (ie, indications, dosages, and date of treatment initiation), or concomitant medications was often not available [31,32,34,35,37].

The presence of chronological criteria (ie, time to onset of ADRs, dechallenge, or rechallenge) was mentioned in only 3 of 11 (27%) studies and varied significantly according to the websites that were analyzed [10,31,36].

In 6 of 11 (55%) studies, the authors verified if the ADRs that were identified in social media were expected or not and compared them with those that had been reported in clinical trials (3/6, 50%) [33,36,37], in pharmacovigilance databases (2/6, 33%) [30,35], or in the studied drugs’ Summary of Product Characteristics (SPC) (1/6, 17%) [31].

Out of 11 studies, 2 (18%) reported using a standard terminology—Medical Dictionary for Regulatory Activities (MedDRA) [30]—or Problem Intervention Documentation (PI-Doc) [36], a German classification system, for coding the ADRs or AEs that were reported in social media. Freifeld et al [30] found that the AEs/ADRs identified in social media had similar profiles to those that were spontaneously reported through official channels, whereas Pages et al described the qualitative differences between the data sources [35].

Some studies identified potential unexpected ADRs [31,35,37] or unexpected frequencies of known ADRs [33,36]. Furthermore, Abou Taam et al retrospectively identified one case of severe valvulopathy 7 months before benfluorex was withdrawn because of this toxicity [28]. In their study, Butt et al compared patients’ unsolicited Internet descriptions of severe cutaneous ADRs with experiences that had been previously collected in face-to-face interviews of survivors of these ADRs [29]. The authors identified new themes from Internet narratives, including fears and concerns of patients who had experienced the condition. According to the authors, patients also reported on more sensitive issues, such as sexual dysfunction, on the Internet rather than in face-to-face interviews.

The ADRs and AEs reported in social media were often less serious than those that were spontaneously reported through official channels [31,35], but they had impaired the patients’ quality of life and their adherence to treatment [30,33,34]. Patients' comments were also related to complications from the ADRs, contraindications, drug-drug and food-drug interactions, and storage of drugs [37]. Users shared their experiences with individuals who were taking the same drug or had had similar adverse events, or with health care providers to obtain information or advice.

Abou Taam et al evaluated the impact of media coverage on benfluorex’s withdrawal in France by analyzing patients' comments at three periods: before, during, and after the withdrawal of the drug. They found messages reflecting anxiety, anger, and other feelings, with drastic changes in consumers' perceptions following media coverage [28].

### Theme 2: Extraction

#### Overview

The 13 studies that were selected for the review had been recently published—2010 for the oldest [42].

**Figure 3:**
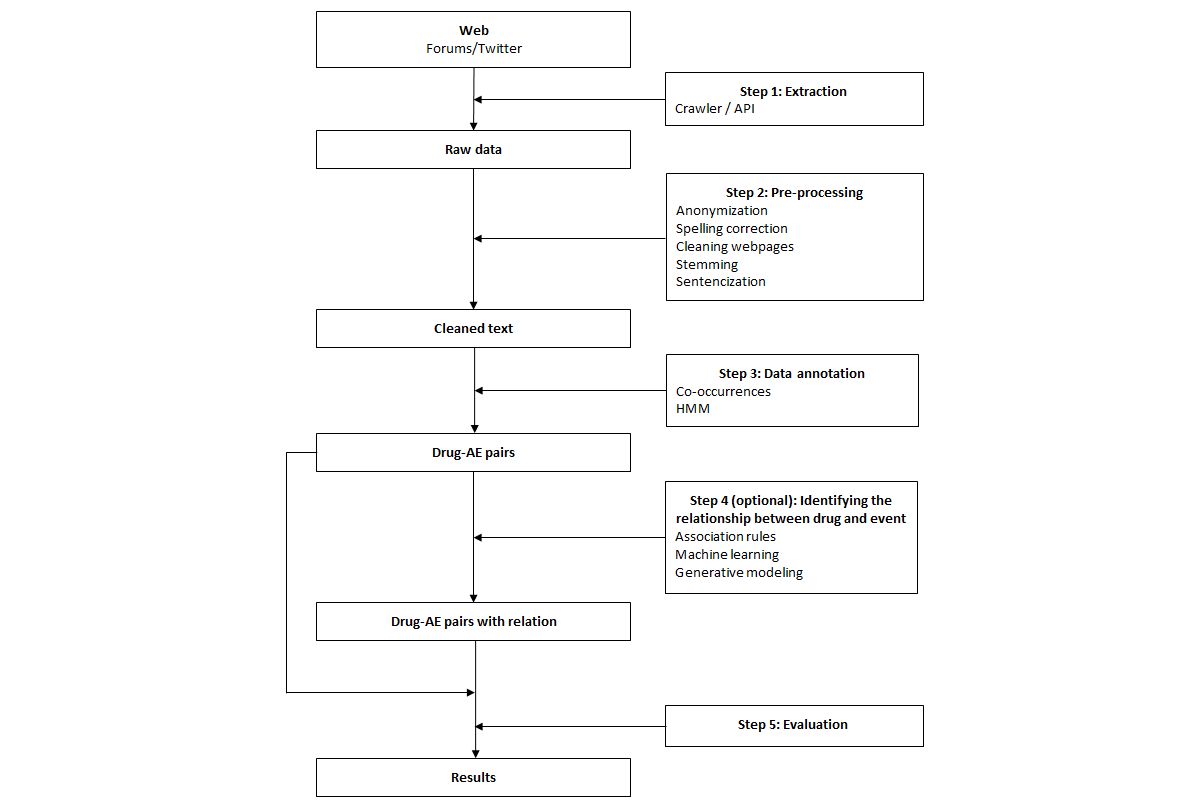
Main steps for extraction of adverse drug reactions (ADRs) from social media.

Figure 3 shows a synthesis of the complete steps and presents five distinct parts: (1) data extraction, (2) preprocessing, (3) data annotation, (4) identifying the relationship between drug and event, and (5) results evaluation.

Multimedia Appendix 3 summarizes the use of these different steps in the papers.

#### Choice of the Source

The main data source was forum discussions in 12 studies out of 13 (92%) [34,35,37,38,40-44,47-49]. Out of the 13 studies, 1 (8%) [39] was about extracting narratives from Tweets.

Each study examined narratives that had been written in English, except for that of Hadzi-Puric and Grmusa [40]. The data volume was heterogeneous and varied from millions of messages [41] or billions of Tweets [39] to a more limited number of messages, such as the 1290 messages included in the study by Hadzi-Puric and Grmusa [40].

The list of studied drugs was also heterogeneous. Out of the 13 selected studies, 11 (85%) focused on a limited number of drugs, such as lipid-modifying drugs in Li [43]. The other studies aimed at detecting signals of a large number of drugs, as in Liu and Chen’s work [44], which considered all of the drugs from the Unified Modeling Language System (UMLS) and the US Food and Drug Administration's (FDA) Adverse Drug Event Reporting System (FAERS).

#### Data Extraction

The operating method to extract data from Web forums and social media depended on the nature of the source. For Web forums, 8 of 13 (62%) articles used an adapted Web crawler to collect Web pages, and then a Web scraper to extract the messages that were embedded in these Web pages [38,42-44,46-49].

Web scraping can be done through two approaches: (1) by taking the whole code of the page and cleaning it by eliminating the HTML tags and other unwanted elements or (2) by targeting the patients' messages using the HTML structure. The first approach was chosen by Benton [38]. In this work, approximately 48% of the tokens, defined as strings of characters delimited by whitespace in the original HTML pages, were retained to generate the corpus.

When the source was Twitter [39], specific application programming interfaces (APIs) were available for extracting data. These APIs provided some structured information, such as the date of the message or the pseudonym of the author, which are benefits to data quality, but the narratives still had to be processed with NLP.

#### Preprocessing Data

Using raw data extracted from social media or Web forums was not straightforward. “Preprocessing” the data was necessary and consisted, for example, of clarifying abbreviations and checking spelling mistakes.

As shown in Table 4, a number of types of transformations were performed on the extracted data.

**Table 4:** Transformations performed on the extracted data.

Transformation	Rationale and methods
Anonymization	Anonymization is required to remove patients’ personal data to comply with medical confidentiality. Benton’s team trained a classifier to determine if a token had to be anonymized or not [38]. Liu and Chen, only, did not extract the author pseudonyms [44], but they did not apply anonymization to the narratives.
Spelling correction	To maximize the detection of information in the corpus, spelling mistakes and typing errors that are common in texts extracted from social networks have to be corrected. The analyzed texts were extracted from social networks or public forums and included many abbreviations and typing errors. Li [43] applied this method to medical words that were often misspelled in messages.
Cleaning Web pages	Web pages consist of hundreds of tags that are invisible to users. When the crawler extracted a complete Web page code, a cleaning step was necessary to refine the content, as with Benton et al [38] and Liu and Chen [44].
Stemming	Reducing inflected words to their root helps to detect different forms of a word. This process reduces words to their word stem, base, or root forms, and these roots were then used for analysis. Different algorithms can be used by the «stemmer» [38,42,45,47,50]. For example, Benton et al [38] and Leaman et al [42] used the «Porter stemmer».
Sentencization/ Tokenization	Breaking the text up into segments of words, sentences, and paragraphs allows for analyzing the sentences and locutions in the corpus. Liu and Chen [44] used sentences at the information extraction level. Similarly, Benton et al [38] and Leaman et al [42] relied on a window of, respectively, 20 and 5 tokens in which the drug and the event co-occurred. Sentencization and tokenization are also documented in Liu and Chen [44], Nikfarjam and Gonzalez [45], and Yeleswarapu et al [50].

#### Annotation

Annotation of the corpus, for instance, identification of adverse events and drugs in messages, was performed in all of the studies reviewed in this theme. Annotation was realized by (1) machine-learning algorithms [39,44] and (2) final statistical evaluation [38-50].

Out of 13 studies, 9 (69%) used standard medical terminology, including Cerner Multum's Drug Lexicon, UMLS, side effect resource (SIDER), Coding Symbols for Thesaurus of Adverse Reaction Terms (COSTART), and MedDRA. Of the 13 studies, 8 (62%) took into account lay language. Among these 13 studies, 7 (54%) used lay vocabulary. Of the 13 that were originally selected, 3 (23%) studies [38,44,48] used a consumer health vocabulary [51], 1 of the 13 (8%) [49] used MedSyn [52], and 3 of the 13 (23%) [40,42,43] used a custom-built vocabulary. Of the 13 studies, 3 (23%) [39,40,44] mapped lay language to medical terminologies using MetaMap [53].

#### Relationships Between Drugs and Events

The relationship between the drug and the medical term was then analyzed. This relationship could have been an indication (ie, the drug was taken to treat the symptom or the disease), a cause (ie, the drug caused the pathology, in this case, an ADR), or a question about a potential causal relationship.

The methods were classified into two categories. The first category corresponds to methods that assessed a relationship between the medication and the event (ie, machine learning, association rules), which were used in 7 studies out of 13 (54%), with machine learning being used in 5 of 13 (38%) publications. When this approach was used, the evaluation was done thanks to cross-validation (3/13, 23%). The second category corresponds to exploratory analysis to identify main safety themes from the corpus of messages (ie, statistically significant co-occurrences) [38,40].

#### Results Evaluation

In the studies that used a computerized approach, the evaluation of results was based on precision (9/13, 69%), recall (9/13, 69%), f-measure (6/13, 46%), accuracy (3/13, 23%), both true- and false-positive rates (1/13, 8%), log-likelihood ratios (1/13, 8%), support (1/13, 8%), confidence (1/13, 8%), leverage (1/13, 8%), and Bayesian confidence propagation neural network (BCPNN) scores and variance (1/13, 8%).

From the initial data volume, authors selected test sets on which they evaluated their systems. The sizes of the test samples were much smaller than the initial volumes of the extracted data. For example, Hadzi-Puric and Grmusa [40] and Li [43] used the whole initial data volume, whereas Yates [49] used only 480 posts out of the initial 400,000 extracted posts.

The pharmacovigilance database that was used for comparison was the FAERS [54] in 4 of the 13 (31%) studies. In 7 other studies out of 13 (54%), the annotators had varying expertise levels—from medical school students to pediatric clinicians and those with PhDs. Benton [38] referred to the tables and notes contained on the drug labels. Overall, only Li [43] did not document the constitution of a new gold standard or the use of an existing standard.

A majority of studies (7/13, 54%) were not only about expected ADRs, but also about discovering relationships between drugs and adverse events that had not been documented on the drug labels or in the literature.

##  Discussion

### Gaps

This scoping review revealed some gaps among the selected studies that could be challenging to fill.

Although some studies that were related to the identification theme concluded that patients’ comments posted in social media contained interesting data for pharmacovigilance (ie, potential unexpected ADRs, patients’ risk perceptions, effects on adherence), they usually failed to accurately assess the completeness, quality, and reliability of these data. We could highlight the near absence of accessible information related to chronology (ie, time to onset, dechallenge, rechallenge) or differential diagnosis that would be necessary to assess the causal relationship between a drug and an AE. Moreover, evaluations of the seriousness and unexpectedness of ADRs was available in only a few studies. Finally, we retrieved no study that took into account exposures during pregnancy and only one study that partly focused on drug-drug or food-drug interactions.

More than the quality of the information shared in social media, issues can be raised about the reliability of this information. Indeed, social media users adopt pseudonyms, which may allow malicious persons to spread false rumors using multiple pseudonyms with limited risk of being identified as the origin of the rumor.

Furthermore, a user can post the same message twice or more on the same forum or on different forums using the same or different pseudonyms with no malevolent intent simply to maximize their chances of obtaining an answer. Consequently, it would be interesting to identify these duplicates. We found only one study in which an algorithm that addressed data redundancy was implemented but not described [40], and removing duplicates was seldom reported as an issue, for example, in Pages et al [35].

Regarding the extraction theme, we identified a set of processing steps that are used to process social media data after the Web crawling step and that could be recommended:

1. Anonymization: this was performed in only 2 studies out of 13 (15%), suggesting that privacy of data was not a major issue for the authors, who considered using pseudonyms to be sufficient for preserving confidentiality; nevertheless, it should be considered in every study that includes personal identifiers.

2. Preprocessing step: checking spelling errors and typographical errors; stemming, sentencization, and tokenization to process social media data.

3. Annotation and use of existing medical terminology.

Because none of the selected articles reported on a method that encompassed all of the steps we considered key, we assume that refining current methods and tools is desirable to improve the quality of processed data.

We also noticed that implementation did not follow a generic approach, which would be necessary for easily adding new sites or data sources. This is understandable in the context of a research project, but genericity should be addressed if more sites are intended to be included in the general pharmacovigilance process.

Finally, from the studies returned by our citation database queries, no study used comments on video-sharing websites as a source of data.

### Limitations

The methodology has at least two limitations. First, when we constituted the research team, we were not exhaustive regarding the stakeholders we included in this review. For example, we lack stakeholders from regulatory agencies, from the pharmaceutical industry, and from patients or patient associations. The second limitation relates to the citation searches. We limited ourselves to PubMed and Embase. Although both of these resources offer a wide range of citations, we potentially missed some citations in the field, as illustrated by the fact that we selected two additional articles that were not found by the queries or by screening the citations. Moreover, the query itself was not trivial because the field is still a new research area. Finally, by using PubMed and Embase, we could not find any analyses of ADRs using social media that were conducted confidentially within company safety departments.

### Perspectives

Emerging evidence on the effectiveness of social media for surveillance suggests that mining messages posted on social media may be helpful for complementing pharmacovigilance systems. Examples of information retrieval from social media have previously been shown in other domains. For instance, it has been demonstrated that Tweets and restaurant reviews might aid in identifying and taking action on localized foodborne illnesses [55-57].

Adverse drug reactions are serious, underreported public health problems with high health and financial costs. A number of authors often described the cases of ADRs reported in social media as insufficiently informative to effectively assess a causal association with the drugs, compared with classical reporting in which quality criteria are available [58,59]. Moreover, extracting ADRs from social media presents specific technical constraints, given the unstructured information, compared with electronic health records or pharmacovigilance databases. Finally, spelling errors and patients’ expressions [60,61] make extraction even more difficult.

However, our study confirms that there is a sufficient volume of data on pharmacovigilance in social media to work with and that quantity may eventually support the pharmacovigilance process despite variable quality. Whereas some websites may collect huge amounts of poorly documented posts, others such as PatientsLikeMe [60] collect very complete and high-quality data on drug treatments and, therefore, present very interesting possibilities for improving our knowledge on ADRs based on reliable information. It is thus necessary to further evaluate the quality of the different websites to fulfill the expectations of a new data source for pharmacovigilance.

Among the conceivable solutions for increasing reliability, we can suggest the use of comments’ metadata (eg, pseudonym, date, and eventually the location given in the profile) to detect duplicated posts from the same author.

Indeed, the objective is to use social media as an additional source of data to expedite signals of potential ADRs. Local pharmacovigilance departments nationwide collect data on adverse events to track cases and interpret data for surveillance. Social media may help to detect the misuse or abuse (including overdose) of drugs [62,63] and adverse effects that would otherwise go unreported (eg, ADRs that are not serious but can impair the patients’ quality of life and the adherence to treatment).

In order to verify the reliability of data retrieved online, comparison of this data with established sources, like FAERS or SIDER, as realized by several authors to derive reference material, can also be useful to detect new knowledge and improve quality of documentation of already described ADRs.

Social media may also provide new information on polypharmacy in real life, especially on the concomitant use of prescription drugs and self-medication drugs, and its consequences for patients, such as drug-drug interactions.

Nevertheless, it is necessary to verify how this new data source could be integrated into regular pharmacovigilance systems, with the aim of detecting, verifying, or validating signals.

Moreover, a number of authors highlighted the necessity of considering the context associated with the drug prescription, including whether any ADRs have been described in the media or discussed by regulatory agencies, to interpret the findings. Through the example of benfluorex, Abou Taam et al [28] analyzed narratives that were posted on French websites and reported drastic changes in consumers’ risk perceptions following media coverage. As such, social media may be analyzed to assess consumers’ behaviors and their risk perceptions and, finally, guide public communication campaigns.

Finally, a broader use of the Internet may include additional sources, such as soliciting reporting studies [64] we excluded, crowdsourcing [65] that may be complementary to social media, or Web search queries [66].

### Conclusions

We conducted a scoping review to explore the potential interest in social media as a new source of data in pharmacovigilance and to define the methods for extracting data from this source. The exploratory aspect of the scoping review helped to give us an overview of this field, and this was a mandatory first step when we began our own work in the field. We are currently developing methods and tools within the Adverse Drug Reactions from Patient Reports In Social Media (ADR-PRISM) and *Vigilance dans les Forums sur le Médicament* (Vigi4MED) projects to collect data from social media and to evaluate the data’s potential interest for pharmacovigilance. This scoping review was beneficial for identifying gaps in previous studies and designing our work plan.

Among the studies that were related to extraction, the oldest one was published in 2010, which shows that this field is still new and suggests that we can expect numerous further developments and improvements to come.

Finally, it appears that there are still outstanding questions about the data collected from social media and that there is sufficient room for improving extraction systems. Depending on the measured characteristics of social media as a new data source for pharmacovigilance and the headway in extracting ADRs, pharmacovigilants will have to define the role of social media in the classical pharmacovigilance system.

## Appendix

Multimedia Appendix 1 Characteristics of included studies from theme 1 (identification).

Multimedia Appendix 2 Characteristics of included studies from theme 2 (extraction).

Multimedia Appendix 3 Preprocessing transformations and analyses used in theme 2 (extraction) studies.

## Abbreviations

ab,ti: abstract, title
ADE: adverse drug event
ADR: adverse drug reaction
ADR-PRISM: Adverse Drug Reactions from Patient Reports In Social Media
AE: adverse event
ANSM: the French drug safety agency (French acronym of Agence Nationale de Sécurité du Médicament et des Produits de Santé)
AP-HP: Assistance Publique-Hôpitaux de Paris
API: application programming interface
BCPNN: Bayesian confidence propagation neural network
CHU: University Hospital Center (French acronym of Centre Hospitalier Universitaire)
COSTART: Coding Symbols for Thesaurus of Adverse Reaction Terms
DGE: block grants for investment expenditures (French acronym of Direction Générale des Entreprises)
FAERS: FDA Adverse Drug Event Reporting System
FDA: Food and Drug Administration
FUI: French acronym of Fond Unique Interministériel
HEGP: Hôpital Européen Georges-Pompidou
INSERM: the French National Institute for Health and Medical Research (French acronym of Institut National de la Santé et de la Recherche Médicale)
LIMICS: Laboratoire d'Informatique Médicale et d'Ingénieurie des Connaissances en e-Santé
MedDRA: Medical Dictionary for Regulatory Activities
MeSH: Medical Subject Heading
NLP: natural language processing
PI-Doc: Problem Intervention Documentation
SIDER: side effect resource
SPC: Summary of Product Characteristics
TIAB: title and abstract
SSPIM: Department of Public Health and Medical Informatics (French acronym of Service de Santé Publique et de l’Information Médicale)
UMLS: Unified Modeling Language System
UMR_S: Unité Mixte de Recherche en Santé
UPMC: University of Pierre and Marie Curie
Vigi4MED: French acronym of Vigilance dans les Forums sur le Médicament
